# Maternal and Early Developmental Effects of Perinatal Exposure to Therapeutically Relevant Doses of Common Nonsteroidal Anti‐Inflammatory Drugs in Rats

**DOI:** 10.1002/bdr2.70104

**Published:** 2026-08-02

**Authors:** Sara Tawany Caetano dos Santos, Reggina Lorena Mellado Castro, Luara Magalhães, Giovanna Ferrari Rosalem, Julia Vitoria Francisco de Aquino, Otávio Galassi Vitale, Livia Trippe Nagaoka, Julia Stein, Beatriz de Matos Manoel, Bárbara Campos Jorge, Arielle Cristina Arena

**Affiliations:** ^1^ Department of Cellular and Molecular Biology Institute of Biosciences of Botucatu, UNESP – Paulista State University Botucatu São Paulo State Brazil

**Keywords:** developmental toxicology, maternal toxicity, NSAIDs, perinatal exposure, pregnancy, reproductive development

## Abstract

**Background:**

Nonsteroidal anti‐inflammatory drugs (NSAIDs) are widely used during pregnancy, yet information on their developmental safety during sensitive perinatal windows remains limited. This study evaluated the maternal and early developmental effects of paracetamol/acetaminophen, aspirin, and nimesulide administered at therapeutically relevant doses during late gestation and lactation.

**Methods:**

Pregnant Wistar rats were treated daily by oral gavage with paracetamol/acetaminophen (APAP; 126 mg/kg/day), aspirin (ASA; 5.6 mg/kg/day), or nimesulide (NIM; 11 mg/kg/day) from gestational day 15 to postnatal day 22. Maternal clinical parameters, body weight, food and water intake, organ weights, hematological and biochemical markers, and maternal behavior were evaluated. In F1 offspring, early developmental indicators, anogenital distance, sexual maturation endpoints, reproductive organ weights, and hematological parameters were assessed.

**Results:**

Exposure to the evaluated NSAIDs produced little evidence of overt maternal toxicity. Maternal body weight, food and water intake, gestational length, and most organ weights remained unchanged. Mild hematological and biochemical alterations were observed, particularly in NIM‐treated dams. Maternal behavior was largely preserved, although APAP‐treated dams showed reduced latency to initiate pup grooming. In offspring, anogenital distance and female reproductive maturation were unaffected. Male offspring exposed to APAP showed earlier testicular descent, whereas ASA exposure was associated with increased seminal vesicle weight. These findings were isolated and were not associated with a consistent pattern of reproductive alterations.

**Conclusion:**

Perinatal exposure to therapeutically relevant doses of paracetamol/acetaminophen, aspirin, and nimesulide produced little evidence of overt maternal or developmental toxicity during the evaluated period. Although a limited number of drug‐specific findings were identified, they were isolated and did not indicate a consistent pattern of reproductive toxicity. These results contribute to the preclinical safety assessment of NSAID exposure during pregnancy and lactation and support further studies to determine the functional relevance of these early observations.

## 1 Introduction

Nonsteroidal anti‐inflammatory drugs (NSAIDs) are among the most widely used medications worldwide and are frequently consumed during pregnancy for the treatment of pain, fever, and inflammatory conditions. These drugs exert their therapeutic effects primarily through the inhibition of cyclooxygenase (COX) enzymes, which reduces the synthesis of prostaglandins involved in inflammatory signaling pathways (Black and Hill 2003; Antonucci et al. 2012). Despite their widespread use, concerns have been raised regarding the safety of NSAID exposure during pregnancy, as prostaglandins play essential roles in multiple physiological processes involved in maternal adaptation and fetal development (Pinheiro and Stika 2020).

During gestation, profound physiological and hormonal changes modify the pharmacokinetic and pharmacodynamic profiles of many drugs. These alterations may facilitate the placental transfer of lipophilic compounds, allowing pharmacological agents to reach the fetal environment during critical windows of development (Syme et al. 2004; Mitchell et al. 2011). Consequently, fetal exposure to commonly used medications may interfere with developmental processes that depend on tightly regulated endocrine, paracrine, and inflammatory signaling pathways (Balin et al. 2020).

Prostaglandins are key mediators in reproductive physiology and participate in implantation, parturition, ovarian function, and neuroendocrine regulation (Antonucci et al. 2012). In addition to these maternal functions, prostaglandins have been implicated in developmental events related to sexual differentiation and reproductive maturation. In particular, prostaglandin E_2_ (PGE_2_) has been shown to participate in the masculinization of the rodent brain through mechanisms involving microglial activation, synaptic plasticity, and neuroendocrine signaling. Therefore, pharmacological inhibition of prostaglandin synthesis during critical developmental periods may influence reproductive development (McCarthy 2020a, 2020b).

Beyond their anti‐inflammatory activity, increasing evidence suggests that some NSAIDs may act as endocrine‐disrupting compounds capable of interfering with hormonal signaling pathways involved in reproductive development. Epidemiological and experimental studies have associated gestational exposure to mild analgesics, particularly paracetamol, with alterations in male reproductive development, including reduced anogenital distance (AGD) and disturbances in pubertal maturation (Kristensen et al. 2011, 2016). Experimental models further indicate that disruption of prostaglandin signaling during critical windows of brain and gonadal development may interfere with steroidogenic pathways and androgen signaling (Bakker and Baum 2008; Stukenborg et al. 2021).

Consistent with these observations, previous studies from our research group have demonstrated that exposure to COX inhibitors can affect reproductive and neuroendocrine‐related outcomes in offspring. Gestational and lactational exposure to ibuprofen was shown to impair hypothalamic sexual differentiation and reproductive parameters in male offspring (Balin et al. 2020). In addition, pre‐pubertal exposure to ibuprofen compromised sperm parameters, testosterone levels, and fertility potential in adult male rats and affected reproductive outcomes in the next generation (Barbosa et al. 2020). Furthermore, perinatal exposure to ibuprofen altered sexual behavior and fertility parameters in female offspring, suggesting long‐term disruption of reproductive function (Jorge et al. 2020).

Importantly, different NSAIDs present distinct pharmacological profiles regarding COX inhibition. Acetylsalicylic acid (aspirin) acts as a non‐selective and irreversible inhibitor of COX enzymes; nimesulide preferentially inhibits COX‐2, and paracetamol/acetaminophen has a more complex mechanism involving central modulation of prostaglandin synthesis (Graham and Scott 2005; Vane and Botting 2003). These pharmacological differences may lead to distinct biological effects during critical windows of development.

Despite growing evidence indicating that COX inhibitors may affect reproductive development, comparatively fewer studies have evaluated different NSAIDs administered at therapeutically relevant doses during the perinatal period, particularly across the window that overlaps with sexual differentiation of the rodent brain (MacLusky and Naftolin 1981; Schwarz and McCarthy 2008; McCarthy 2020a, 2020b). While previous studies from our group have investigated the developmental effects of ibuprofen, it remains unclear whether other commonly used NSAIDs with distinct pharmacological profiles, such as paracetamol/acetaminophen, aspirin, and nimesulide, produce similar early maternal or developmental effects.

Therefore, we hypothesized that perinatal exposure to therapeutically relevant doses of NSAIDs with different COX‐inhibition profiles may induce subtle maternal physiological changes and alter early reproductive developmental markers in offspring. To test this hypothesis, the present study evaluated the effects of paracetamol/acetaminophen, aspirin, and nimesulide exposure from late gestation through lactation on maternal clinical, behavioral, hematological, and biochemical parameters, as well as offspring early development, pubertal markers, reproductive organ weights, and hematological parameters.

## 2 Material and Methods

### 2.1 Animals and Ethical Approval

Wistar male (*n* = 12, 8 weeks, 300 g) and female (*n* = 40, 8 weeks, 200 g) rats were obtained from the animal facility of the Institute of Biosciences of Botucatu, São Paulo State University (UNESP), Brazil. Animals were maintained under controlled environmental conditions (22°C ± 2°C, 12 h light/dark cycle, and 50% relative humidity) with free access to standard phytoestrogen‐free chow (Nuvilab CR1, Nuvital‐PR, Brazil) and water *ad libitum*.

All experimental procedures were conducted in accordance with the Brazilian guidelines for the care and use of laboratory animals established by the National Council for the Control of Animal Experimentation (CONCEA) and followed the ARRIVE guidelines for reporting animal research. The experimental protocol was approved by the Institutional Committee for Ethics in the Use of Animals of São Paulo State University (CEUA‐IBB/UNESP, Botucatu, Brazil; protocol No. 7611070225).

### 2.2 Mating and Experimental Design

**FIGURE 1:**
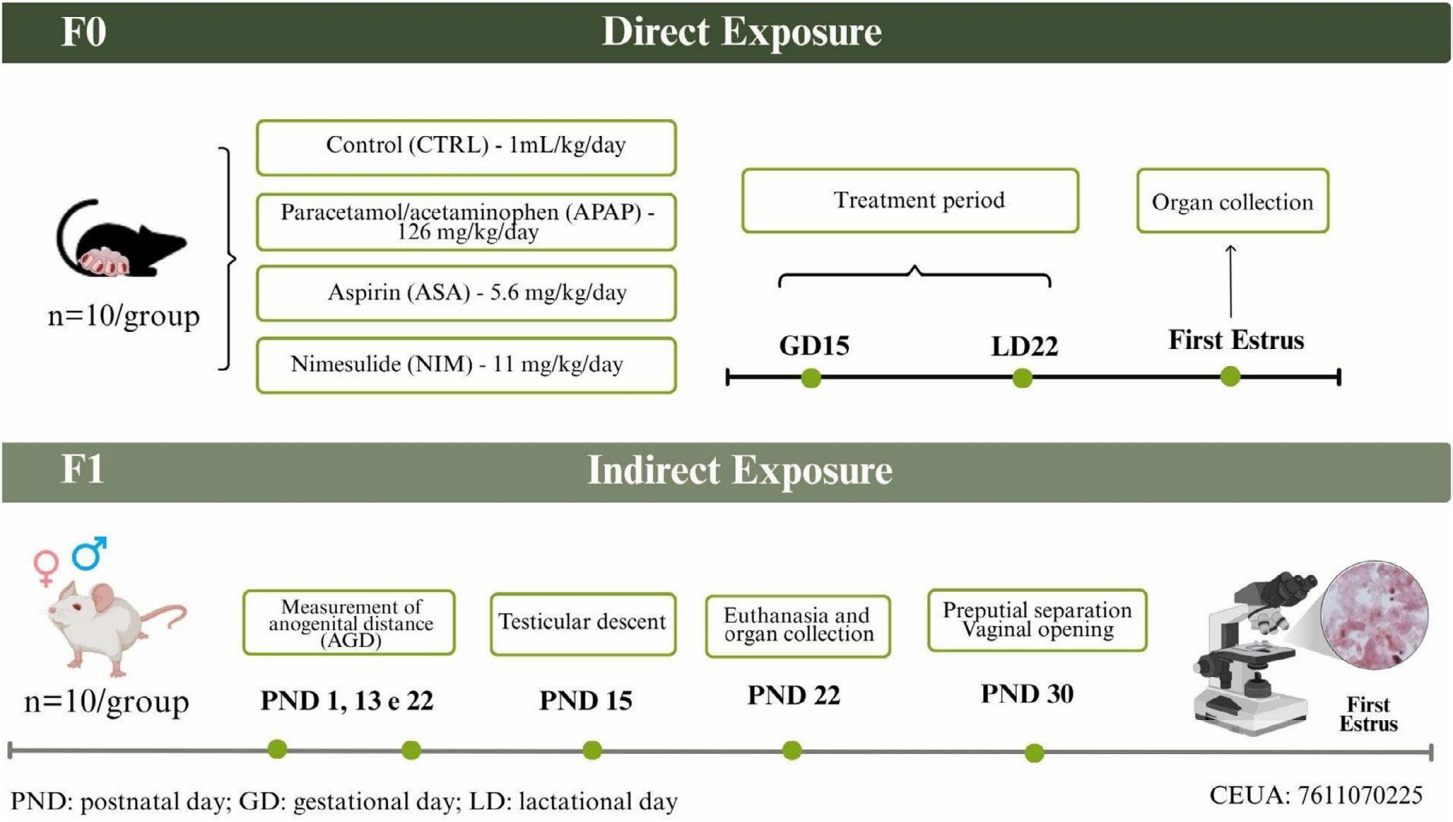
Experimental design.

Adult females were housed overnight with males in a 2:1 ratio for mating. The presence of spermatozoa and cornified epithelial cells (estrus) in the vaginal smear the following morning was considered gestational day (GD) 0. Pregnant females were randomly distributed into four experimental groups (*n* = 10 females/group): (1) Control (CTRL; distilled water, 1 mL/kg/day); (2) Paracetamol/acetaminophen (APAP; 126 mg/kg/day); (3) Acetylsalicylic acid/aspirin (ASA; 5.6 mg/kg/day); and (4) Nimesulide (NIM; 11 mg/kg/day). Treatments were administered once daily by oral gavage from GD15 to postnatal day 22 (PND22), covering late gestation and the lactation period (Figure 1). This exposure window includes critical stages of steroid‐induced sexual differentiation of the male rat brain, which occurs from the last week of gestation until approximately PND10 (MacLusky and Naftolin 1981; Schwarz and McCarthy 2008).

In this experimental design, offspring were exposed to the drugs through placental transfer during gestation and through milk during the lactation period. Treatment was maintained until the end of lactation because this stage is characterized by rapid brain development, including neuronal migration and differentiation, synaptogenesis, glial proliferation, and myelination, representing a critical window of neuronal maturation (Morgane et al. 1993; Balin et al. 2020). Maternal physiological parameters, including maternal body weight, clinical signs of toxicity, maternal behavior, and food and water consumption, were monitored throughout gestation and lactation. After birth, on PND1, litter size was standardized to eight pups per litter (four males and four females) whenever possible. Offspring were subsequently evaluated for early developmental markers and reproductive maturation endpoints. To minimize litter effects, one pup per litter was used for each experimental analysis, and the litter was considered the statistical unit for analyses of offspring data.

### 2.3 Drugs and Chemicals

Paracetamol/acetaminophen (98% purity), acetylsalicylic acid (Aspirin, 98% purity), and nimesulide (98% purity) were purchased from Cruz Vermelha Pharmacy (São Paulo, Brazil). All drugs were dissolved in distilled water to the desired concentrations daily before administration by oral gavage.

### 2.4 Dose Selection

Dose selection was based on therapeutic doses commonly used in humans. For paracetamol/acetaminophen, a daily human dose of 2250 mg (750 mg administered three times daily) was considered, corresponding to 126 mg/kg/day in rats. For aspirin, the dose was based on the low‐dose regimen commonly prescribed during pregnancy for the prevention of preeclampsia (100 mg/day), corresponding to 5.6 mg/kg/day in rats. For nimesulide, the human therapeutic regimen of 100 mg twice daily was used for dose calculation, resulting in a rat dose of 11 mg/kg/day. The doses were determined based on therapeutic concentrations recommended for humans and converted to animal doses according to the following formula: human dose (mg/kg BW) = animal dose (mg/kg BW) × (animal body weight/human body weight)^0.25 (US EPA 2011), considering an average human body weight of 70 kg and a rat body weight of 0.30 kg. These doses correspond to therapeutically relevant exposures and have been previously used in experimental studies evaluating the developmental effects of NSAIDs. The drugs were evaluated individually because the aim of the study was to identify drug‐specific maternal and developmental effects associated with distinct pharmacological profiles.

### 2.5 Maternal Evaluation

#### 2.5.1 Monitoring of Maternal Physiological Parameters

During NSAID exposure, pregnant dams (*n* = 10/group) were monitored for food and water consumption as well as body weight gain on alternate days for dose adjustment. In addition, clinical signs of toxicity were recorded throughout the exposure period, including diarrhea, piloerection, bleeding, abnormal respiration, tremors, convulsions, alterations in gait, posture, and response to handling.

#### 2.5.2 Maternal Behavior

Maternal behavior was evaluated on lactational day 5 (LD5). This time point was chosen because maternal motivation is well‐established and pups are sufficiently developed to be retrieved (Champagne et al. 2003). Dams were separated from their pups for 30 min, after which the nest was disrupted, and pups were distributed randomly throughout the cage. The dams were then reintroduced into the cage, and maternal behavior was observed for 30 min. The following parameters were recorded: latency to initiate pup grooming, time required for pup grouping, time spent licking pups, time spent without pup interaction, and nest quality score. Nest quality was scored on a scale from 0 to 3, where: 0 = no nest; 1 = poorly organized nest without walls; 2 = organized nest with low to moderate walls; and 3 = well‐organized nest with high walls.

#### 2.5.3 Organ and Blood Collection

At the end of the lactation period, and after milk collection, dams were euthanized by CO_2_ inhalation during the first estrus for organ and blood collection. The organs (liver, spleen, adrenal glands, thyroid, kidneys, ovaries, and uterus) were collected, dissected, and weighed using a precision analytical balance. Blood samples were obtained by cardiac puncture and divided into two tubes: one containing a clot activator for biochemical analysis and another containing EDTA for hematological analysis.

#### 2.5.4 Hematological and Biochemical Parameters

Blood collected in EDTA tubes was used for hematological analyses, including total leukocyte, erythrocyte, and platelet counts, as well as hemoglobin concentration, hematocrit, and red blood cell distribution width. Analyses were performed using a semi‐automated hematology analyzer (Sysmex KX‐21 N Automated Hematology Analyzer; Kobe, Japan).

Blood samples collected in clot activator tubes were centrifuged at 2400 rpm for 20 min at 4°C to obtain serum. Biochemical parameters were determined using a semi‐automated colorimetric analyzer (BioPlus BIO‐200 Semi‐Automatic Biochemical Analyzer; Bioplus Produtos para Laboratórios Ltda., Barueri, Brazil) with commercial kits (Quibasa‐Bioclin). The following serum parameters were evaluated: albumin, total protein, cholesterol, urea, uric acid, alkaline phosphatase, gamma‐glutamyl transferase (γGT), creatinine, alanine aminotransferase (ALT), and aspartate aminotransferase (AST).

### 2.6 Offspring Evaluation

#### 2.6.1 Early Developmental Assessment

Body weight, AGD, and areola number were evaluated in the offspring. On PND 1, 13, and 22, AGD—defined as the distance between the anus and the genital tubercle—was measured using a digital caliper. AGD is considered a sensitive marker of prenatal androgen action and can be altered by exposure to anti‐androgenic compounds (Thankamony et al. 2016). Body weight was also recorded on these days to evaluate growth and to calculate the relative AGD/body weight ratio. On PND13, the number of areolas was assessed in the pups based on the presence or absence of nipples or skin discoloration around the nipple area. To avoid litter effects, one offspring per litter was used for each analysis.

#### 2.6.2 Vaginal Opening and First Estrus in Female Offspring

From PND30 onward, vaginal opening was evaluated daily in female offspring. Vaginal opening is considered a marker of pubertal onset and is generally associated with the establishment of estrous cyclicity and the first ovulation (Vidal 2017). The day of complete vaginal opening was recorded, and females were weighed on that day. After vaginal opening, vaginal smears were collected daily to determine the day of first estrus, characterized by the predominance of cornified epithelial cells (Marcondes et al. 2002).

#### 2.6.3 Testicular Descent and Preputial Separation in Male Offspring

From PND15, testicular descent—one of the earliest indicators of male reproductive development—was evaluated daily in male offspring through visual inspection and gentle palpation of the scrotal region to verify the presence of testes within the scrotum. From PND30, preputial separation (a marker of pubertal onset in male rodents) was also assessed daily. Animals were examined visually and, with gentle manipulation of the external genitalia, the separation of the prepuce from the glans penis was evaluated. The day on which complete separation was observed was recorded, and males were weighed on that day.

#### 2.6.4 Organ and Blood Collection

On PND22, one male and one female offspring per litter were euthanized for organ collection and hematological analysis. Offspring blood samples were collected and processed for hematological analyses using the same procedure described above for maternal blood samples. The remaining offspring were maintained for the continuation of reproductive developmental assessments.

### 2.7 Statistical Analysis

The obtained data were first tested for normality using the Shapiro–Wilk test to classify them as parametric or nonparametric. Parametric data were analyzed using one‐way analysis of variance (ANOVA) followed by Dunnett's post hoc test, and results were expressed as mean ± standard error of the mean (SEM). Nonparametric data were analyzed using the Kruskal–Wallis test followed by Dunn's post hoc test, and results were expressed as median with interquartile range (Q1–Q3). Differences were considered statistically significant when *p* < 0.05. Statistical analyses were performed using GraphPad InStat software (version 7.02). Sample size was determined based on previous studies from our group and was sufficient to detect biologically relevant differences (power = 0.80, *α* = 0.05).

## 3 Results

### 3.1 Maternal Physiological Parameters

Maternal physiological parameters were monitored throughout gestation and lactation to evaluate possible systemic effects of NSAID exposure. No overt clinical signs of toxicity were observed in any treated group during the experimental period. Maternal body weight gain during gestation and lactation did not differ significantly among the experimental groups. Similarly, food and water consumption remained comparable between control and treated dams throughout the treatment period (Table 1). No premature delivery or evident delay in parturition was observed in any experimental group, and all dams delivered within the expected gestational period for Wistar rats.

**TABLE 1:** Body weight, water consumption and food intake of dams treated with NSAIDs during gestation and lactation.

Parameters	Experimental groups			
	CTRL	APAP	ASA	NIM
Initial body weight (g)	304.0 ± 7.70	304.5 ± 6.82	295.0 ± 8.11	303.0 ± 7.33
Final body weight (g)	305.7 ± 8.94	305.6 ± 5.95	298.3 ± 6.66	306.2 ± 9.68
Body weight gain (%)	0.48 ± 0.77	0.45 ± 1.19	1.30 ± 1.28	0.52 ± 1.56
Food intake (g/day)	38.05 ± 2.29	38.46 ± 2.52	38.72 ± 2.16	38.86 ± 2.44
Water intake (mL/day)	59.72 ± 3.51	58.3 ± 3.86	64.31 ± 4.55	65.47 ± 3.51

*Note:* Values are expressed as mean ± standard error of the mean (SEM). *n* = 10 dams/group. One‐way ANOVA followed by Dunnett's post hoc test.

Abbreviations: APAP, paracetamol/acetaminophen; ASA, aspirin; CTRL, control; NIM, nimesulide.

Only the NIM‐treated group showed a reduction in the relative weight of the right ovary compared with the control group. However, this alteration was not accompanied by consistent changes in the contralateral ovary or total ovarian weight. No statistically significant changes in maternal organ weights were observed in the other treated groups (Table 2).

**TABLE 2:** Relative organ weights (g/100 g body weight) of dams treated with NSAIDs during gestation and lactation.

Parameters	Experimental groups			
	CTRL	APAP	ASA	NIM
Body weight (g)	283.2 ± 5.47	274.9 ± 8.88	278.6 ± 6.09	294.1 ± 3.64
Right ovary	0.017 ± 0.001	0.016 ± 0.003	0.015 ± 0.003	0.013 ± 0.001*
Total ovaries	0.033 ± 0.002	0.033 ± 0.002	0.030 ± 0.001	0.030 ± 0.004
Uterus	0.163 ± 0.008	0.164 ± 0.008	0.140 ± 0.009	0.134 ± 0.008
Spleen	0.192 ± 0.008	0.194 ± 0.008	0.186 ± 0.008	0.191 ± 0.009
Adrenal gland	0.015 ± 0.000	0.016 ± 0.000	0.016 ± 0.000	0.016 ± 0.000
Kidney	0.455 ± 0.019	0.450 ± 0.011	0.463 ± 0.011	0.447 ± 0.007
Liver	5.063 ± 0.393	4.419 ± 0.165	5.379 ± 0.081	5.408 ± 0.160
Thyroid	0.006 ± 0.000	0.006 ± 0.000	0.005 ± 0.000	0.005 ± 0.000

*Note:* Values are expressed as mean ± standard error of the mean (SEM). *n* = 10 dams/group. One‐way ANOVA followed by Dunnett's post hoc test.

Abbreviations: APAP, paracetamol/acetaminophen; ASA, aspirin; CTRL, control; NIM, nimesulide.

* *p* < 0.05 compared with the control group.

**FIGURE 2:**
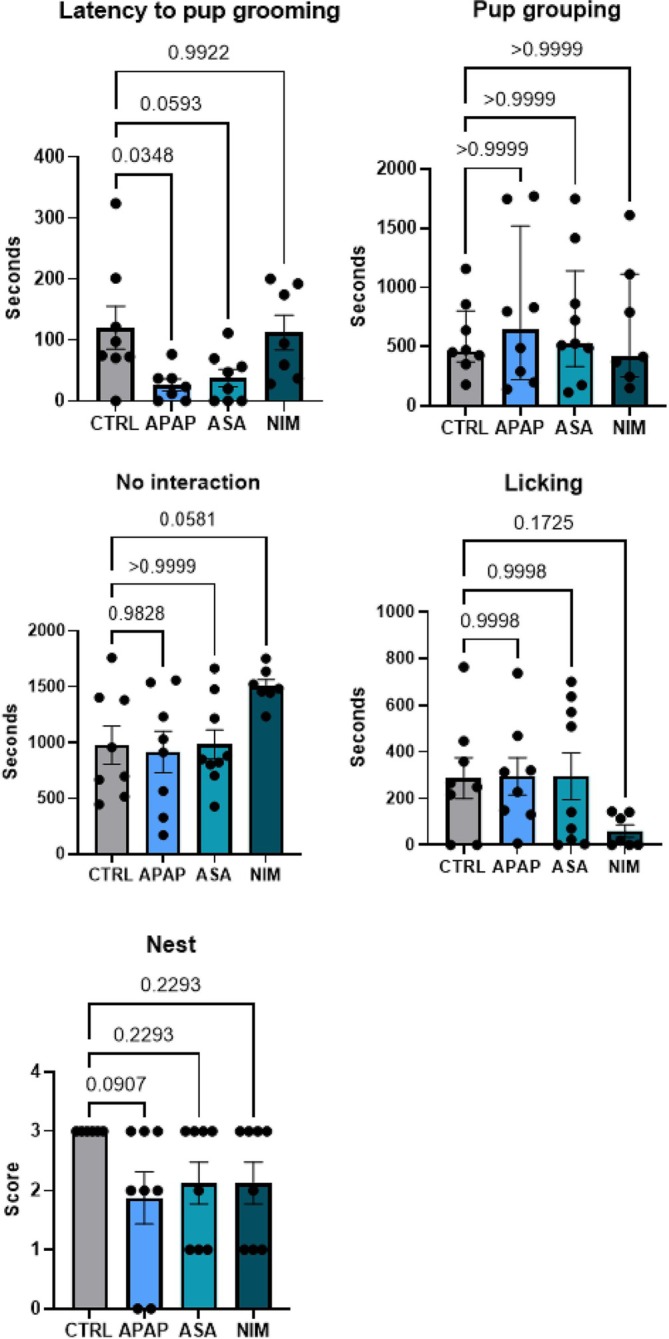
Maternal behavior of lactating rats exposed to NSAIDs during gestation and lactation. Values are expressed as mean ± SEM. One‐way ANOVA followed by Dunnett's post hoc test. Nonparametric data were analyzed by Kruskal–Wallis followed by Dunn's post hoc test. *n* = 10 dams/group. Exact *p*‐values are shown above the brackets. Differences were considered statistically significant at *p* < 0.05 compared with the control group. CTRL. Maternal behavior was evaluated on lactational day 5. The figure shows latency to initiate pup grooming, time required for pup grouping, time spent without interaction, time spent licking pups, and nest quality score. APAP, paracetamol/acetaminophen; ASA, aspirin; CTRL, control; NIM, nimesulide.

Maternal behavior was assessed on lactational day 5. Dams treated with paracetamol/acetaminophen showed a reduced latency to initiate pup grooming compared with controls. However, no statistically significant differences were observed in pup grouping, licking behavior, no‐interaction time, or nest quality scores among the experimental groups (Figure 2).

Analysis of hematological parameters revealed that only the NIM‐treated group showed alterations, characterized by a decrease in hematocrit values and an increase in mean corpuscular volume (MCV) and mean corpuscular hemoglobin (MCH), whereas the other groups did not exhibit significant changes. Regarding biochemical parameters, dams treated with ASA and NIM showed increased levels of aspartate aminotransferase (AST) and urea. Additionally, only the NIM‐treated group exhibited elevated serum levels of alkaline phosphatase (Table 3).

**TABLE 3:** Hematological and biochemical parameters of dams treated with NSAIDs during gestation and lactation.

Parameters	Experimental groups			
	CTRL	APAP	ASA	NIM
*Hematological*				
Platelets (10^3^/μL)	1085 ± 65.33	1046 ± 63.85	1146 ± 35.25	1020 ± 55.97
Erythrocytes (10⁶/μL)	8.22 ± 0.18	8.31 ± 0.08	8.15 ± 0.08	7.51 ± 0.12*
Hematocrit (%)	47.83 ± 1.15	48.61 ± 0.73	48.22 ± 0.87	44.35 ± 1.2*
#Leukocytes (10^3^/μL)	8.2 (7.6–10.3)	7.4 (6.3–8.2)	7.9 (7.4–10.5)	7.8 (7.0–10.3)
Hemoglobin (g/dL)	15.41 ± 0.32	16.0 ± 0.21	15.62 ± 0.21	14.58 ± 0.45
MCV (fL)	58.18 ± 0.79	59.01 ± 0.34	59.12 ± 0.74	61.24 ± 1.37*
MCH (pg)	18.76 ± 0.23	19.23 ± 0.12	19.13 ± 0.20	20.10 ± 0.42*
MCHC (g/dL)	32.23 ± 0.27	32.59 ± 0.13	32.42 ± 0.36	32.84 ± 0.20
Lymphocytes (%)	74.13 ± 1.58	72.88 ± 2.32	70.67 ± 3.06	79.0 ± 1.09
Neutrophils (%)	22.0 ± 1.73	23.5 ± 2.06	25.5 ± 2.74	16.83 ± 1.45
Monocytes (%)	2.25 ± 0.36	1.75 ± 0.49	2.33 ± 0.56	2.33 ± 0.42
*Biochemicals*				
AST (U/dL)	96.86 ± 14.48	96.57 ± 6.68	195.5 ± 17.92*	182.3 ± 21.86*
ALT (U/L)	80.5 ± 13.22	50.75 ± 6.51	65.75 ± 11.54	82.0 ± 19.74
Total protein (g/dL)	4.54 ± 0.52	3.26 ± 0.42	4.03 ± 0.42	3.88 ± 0.39
Albumin (g/dL)	3.95 ± 0.56	3.4 ± 0.34	3.69 ± 0.47	3.25 ± 0.30
Urea (mg/dL)	57.43 ± 2.53	51.00 ± 5.23	77.00 ± 2.0*	74.67 ± 2.55*
Creatinine (mg/dL)	0.48 ± 0.09	0.48 ± 0.06	0.59 ± 0.02	0.5 ± 0.05
Calcium (mg/dL)	34.01 ± 3.07	26.1 ± 2.12	37.31 ± 2.53	36.3 ± 2.89
Cholesterol (mg/dL)	191.4 ± 43.23	101.8 ± 12.87	146.0 ± 15.02	138.5 ± 22.66
GGT (U/L)	5.0 ± 1.46	2.5 ± 1.35	2.5 ± 0.62	1.33 ± 0.84
Uric acid (mg/dL)	12.58 ± 2.47	9.09 ± 1.47	12.14 ± 2.25	7.78 ± 0.9
ALP (U/L)	289.0 ± 58.76	319.8 ± 98.59	480.0 ± 80.88	647.8 ± 124.4

*Note:* Values are expressed as mean ± standard error of the mean (SEM). *n* = 10 dams/group. One‐way ANOVA followed by Dunnett's post hoc test. Values marked with # are expressed as median and interquartile range (Q1–Q3) and were analyzed by Kruskal–Wallis followed by Dunn's post hoc test.

Abbreviations: ALP, alkaline phosphatase; ALT, alanine aminotransferase; APAP, paracetamol/acetaminophen; ASA, aspirin; AST, aspartate aminotransferase; CTRL, control; GGT, gamma‐glutamyl transferase; MCH, mean corpuscular hemoglobin; MCHC, mean corpuscular hemoglobin concentration; MCV, mean corpuscular volume; NIM, nimesulide.

* *p* < 0.05 compared with the control group.

### 3.2 Offspring Evaluation

When analyzing the early development of the F1 offspring, relative AGD did not show relevant variations among the experimental groups in either males or females on PND 1, 13, and 22 (Table 4).

**TABLE 4:** Relative anogenital distance of male and female offspring treated with NSAIDs during gestation and lactation.

Parameters	Experimental groups			
	CTRL	APAP	ASA	NIM
**Males**				
*PND 1*				
Relative AGD (mm/g)	2.51 ± 0.04	2.57 ± 0.04	2.55 ± 0.05	2.63 ± 0.07
Body weight (g)	10.16 ± 0.21	10.14 ± 0.38	10.44 ± 0.34	10.48 ± 0.17
*PND 13*				
Relative AGD (mm/g)	3.62 ± 0.07	3.80 ± 0.04	3.59 ± 0.07	3.79 ± 0.06
Body weight (g)	26.63 ± 0.63	26.68 ± 1.28	27.41 ± 0.92	27.38 ± 1.02
*PND 22*				
Relative AGD (mm/g)	5.19 ± 0.01	5.09 ± 0.06	5.09 ± 0.06	5.13 ± 0.08
Body weight (g)	49.91 ± 1.08	51.22 ± 2.57	50.37 ± 1.56	51.82 ± 2.28
**Females**				
*PND 1*				
Relative AGD (mm/g)	1.45 ± 0.03	1.42 ± 0.04	1.46 ± 0.04	1.51 ± 0.03
Body weight (g)	9.84 ± 0.22	10.15 ± 0.36	10.16 ± 0.30	10.25 ± 0.23
*PND 13*				
Relative AGD (mm/g)	2.70 ± 0.06	2.69 ± 0.05	2.71 ± 0.04	2.64 ± 0.04
Body weight (g)	25.59 ± 0.64	25.96 ± 1.12	26.72 ± 0.74	26.02 ± 0.80
*PND 22*				
Relative AGD (mm/g)	3.38 ± 0.06	3.45 ± 0.03	3.45 ± 0.02	3.49 ± 0.04
Body weight (g)	49.56 ± 1.72	50.34 ± 2.12	49.0 ± 1.51	49.23 ± 1.89

*Note:* Values are expressed as mean ± standard error of the mean (SEM). The litter was used as the statistical unit of comparison; *n* = 10 litters/group. One‐way ANOVA followed by Dunnett's post hoc test. Anogenital distance was evaluated on postnatal days (PND) 1, 13, and 22. Relative anogenital distance was calculated as the ratio between anogenital distance and body weight.

Abbreviations: AGD, anogenital distance; APAP, paracetamol/acetaminophen; ASA, aspirin; CTRL, control; NIM, nimesulide.

**FIGURE 3:**
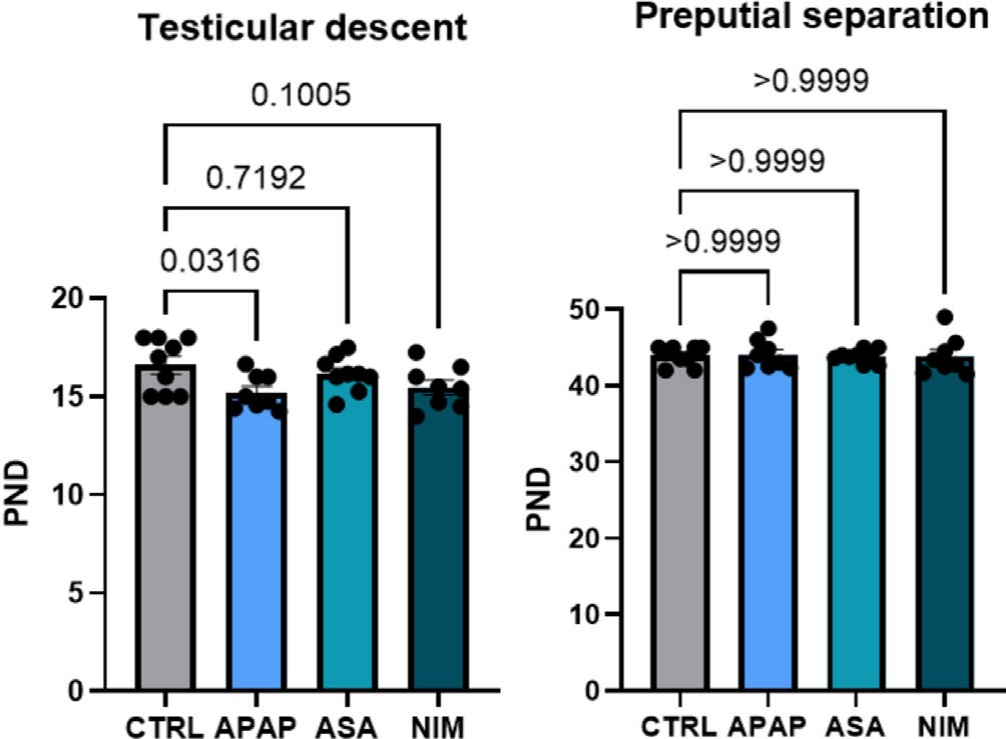
Age at testicular descent and preputial separation in male offspring exposed to NSAIDs during gestation and lactation. The age at each developmental milestone is expressed in postnatal days (PND). Values are expressed as mean ± SEM. The litter was used as the statistical unit of comparison; *n* = 10 litters/group. One‐way ANOVA followed by Dunnett's post hoc test. Differences were considered statistically significant at *p* < 0.05 compared with the control group. APAP, paracetamol/acetaminophen; ASA, aspirin; CTRL, control; NIM, nimesulide.

Regarding male reproductive development, offspring from the APAP‐treated group showed an earlier age at testicular descent compared with controls [CTRL: 16.70 ± 0.45 PND; APAP: 15.10 ± 0.31* PND; ASA: 16.17 ± 0.30 PND; NIM: 15.48 ± 0.38 PND; *p < 0.05]. No significant differences were observed in the ASA‐ or NIM‐exposed groups compared with controls. Preputial separation occurred at a similar age among all experimental groups (Figure 3). In female offspring, no significant differences were observed between treated groups and controls in relation to early sexual development or the onset of puberty, typically indicated by the first estrus (Table 5).

**TABLE 5:** Early sexual development and pubertal onset in female offspring treated with NSAIDs during gestation and lactation.

Parameters	Experimental groups			
	CTRL	APAP	ASA	NIM
Vaginal opening (days)	33.16 ± 0.45	33.13 ± 0.54	33.06 ± 0.70	33.51 ± 0.84
Body weight at vaginal opening (g)	113.5 ± 2.98	108.2 ± 2.52	110.3 ± 2.18	102.5 ± 4.01
First estrus (days)	36.07 ± 1.15	34.55 ± 0.64	36.72 ± 0.78	35.76 ± 1.17
Body weight at first estrus (g)	124.6 ± 2.46	119.0 ± 4.58	128.3 ± 3.30	124.0 ± 5.17

*Note:* Age at vaginal opening and first estrus is expressed in postnatal days (PND). Values are expressed as mean ± standard error of the mean (SEM). The litter was used as the statistical unit of comparison; *n* = 10 litters/group. One‐way ANOVA followed by Dunnett's post hoc test.

Abbreviations: APAP, paracetamol/acetaminophen; ASA, aspirin; CTRL, control; NIM, nimesulide.

At PND22, only males from the ASA‐treated group showed an increase in the relative weight of the seminal vesicle, while the remaining groups presented values similar to those of the control group Table S2. Finally, female offspring did not exhibit significant differences in reproductive or vital organ weights when compared with the control group Table S1. In addition, no significant alterations were observed in the hematological parameters of either male or female offspring exposed to the different NSAIDs when compared to their respective control groups Tables S3 and S4.

## 4 Discussion

The present study evaluated the effects of perinatal exposure to three commonly used NSAIDs with distinct pharmacological profiles, administered at therapeutically relevant doses, on maternal physiology and early reproductive development in rats. Overall, exposure to paracetamol/acetaminophen, aspirin, or nimesulide produced little evidence of overt maternal or developmental toxicity. Most maternal, developmental, and reproductive endpoints remained unchanged. Although a limited number of statistically significant findings were identified, including mild maternal biochemical alterations, reduced latency to initiate pup grooming, earlier testicular descent following paracetamol/acetaminophen exposure, and increased seminal vesicle weight after aspirin exposure, these effects were isolated and were not associated with a consistent pattern of reproductive alterations. Taken together, these findings indicate that, under the experimental conditions employed, the evaluated NSAIDs were generally well tolerated during the perinatal period while identifying a small number of treatment‐related changes that merit further investigation.

Under the experimental conditions employed, maternal exposure to therapeutically relevant doses of the evaluated NSAIDs produced little evidence of systemic toxicity. Body weight gain, food and water consumption, gestational length, and most maternal organ weights remained unchanged, indicating that the treatments were generally well tolerated throughout gestation and lactation. Although a reduction in the relative weight of the right ovary was observed in the nimesulide‐treated group, this change was unilateral and was not accompanied by alterations in the contralateral ovary or total ovarian weight, suggesting that it does not represent consistent ovarian toxicity.

Early postnatal development of the offspring was largely unaffected by perinatal NSAID exposure. Relative AGD, an established marker of prenatal androgen action (Thankamony et al. 2016), remained unchanged in both sexes throughout the evaluation period. Likewise, female reproductive development was preserved, with no significant differences in vaginal opening, first estrus, body weight at pubertal onset, or reproductive organ weights. These findings indicate that exposure to therapeutically relevant doses of the evaluated NSAIDs did not produce widespread disturbances in early reproductive development.

Among the evaluated markers of male reproductive development, only two isolated statistically significant findings were observed. Male offspring exposed to paracetamol/acetaminophen showed earlier testicular descent, whereas aspirin exposure was associated with increased seminal vesicle weight at PND22. However, these findings occurred in isolation and were not accompanied by changes in AGD, preputial separation, testicular weight, or other reproductive endpoints. These results do not indicate a consistent pattern of reproductive toxicity but rather suggest that the evaluated NSAIDs produced limited and drug‐specific effects under the present experimental conditions.

The present findings should also be interpreted in the context of previous studies from our research group. Earlier investigations using ibuprofen demonstrated alterations in hypothalamic sexual differentiation, reproductive behavior, sperm quality, fertility, and reproductive outcomes in subsequent generations (Balin et al. 2020; Barbosa et al. 2020; Jorge et al. 2020). In contrast, the present study evaluated three different NSAIDs with distinct pharmacological profiles and focused specifically on maternal physiology and early developmental endpoints. Therefore, direct comparisons are limited between studies, as differences in the tested compounds, pharmacological properties, experimental design, and evaluated outcomes may account for the distinct findings.

Despite the predominance of negative findings, the present study provides important preclinical information regarding the developmental safety of commonly used NSAIDs administered at therapeutically relevant doses. Comparative studies evaluating different NSAIDs during the perinatal period remain limited, particularly during the developmental window overlapping sexual differentiation of the rodent brain (MacLusky and Naftolin 1981; Schwarz and McCarthy 2008; McCarthy 2020a, 2020b). By directly comparing drugs with distinct COX inhibition profiles under the same experimental conditions, the present study contributes to a more comprehensive understanding of their developmental safety profile. The results indicate that, under the conditions evaluated, these compounds produced little evidence of overt maternal or developmental toxicity while identifying a small number of drug‐specific responses that warrant further investigation.

The present study has some limitations that should be considered when interpreting the findings. First, the assessment was restricted to early developmental endpoints, without follow‐up into adulthood to determine whether the observed changes persist or result in functional reproductive consequences. Second, histological, hormonal, molecular, and fertility analyses were beyond the scope of the present study and therefore mechanistic conclusions cannot be established. Finally, as with any animal model, extrapolation to humans should be made cautiously because interspecies differences in pharmacokinetics, placental transfer, lactational exposure, and developmental timing may influence the observed outcomes (Syme et al. 2004; Pinheiro and Stika 2020; ICH 2020). Consequently, the present findings should not be interpreted as evidence of clinical safety during pregnancy or lactation but rather as preclinical evidence obtained under controlled experimental conditions.

In conclusion, perinatal exposure to therapeutically relevant doses of paracetamol/acetaminophen, aspirin, and nimesulide produced little evidence of overt maternal or developmental toxicity during the evaluated period. Although a limited number of statistically significant, drug‐specific findings were identified, these effects were isolated and were not accompanied by a consistent pattern of reproductive alterations. Nevertheless, the results indicate that subtle biological responses may occur even in the absence of overt toxicity. Overall, these findings contribute to the preclinical safety assessment of NSAID exposure during pregnancy and lactation and provide a rationale for future studies investigating the functional relevance of these isolated early‐life observations.

## Funding

This work was supported by Fundação de Amparo à Pesquisa do Estado de São Paulo (2025/02352‐9).

## Conflicts of Interest

The authors declare no conflicts of interest.

## Supporting information


**Table S1:** Relative organ weights (g/100 g body weight) of female offspring at PND 22.
**Table S2:** Relative organ weights (g/100 g body weight) of male offspring at PND 22.
**Table S3:** Hematological parameters of female offspring at PND22.
**Table S4:** Hematological parameters of male offspring at PND22.

## Data Availability

The data that support the findings of this study are available on request from the corresponding author. The data are not publicly available due to privacy or ethical restrictions.
