# Supplementary material for: Maternal and Early Developmental Effects of Perinatal Exposure to Therapeutically Relevant Doses of Common Nonsteroidal Anti‐Inflammatory Drugs in Rats
Source: Birth Defects Res. 2026 Aug 2;118(8):e70104. doi: 10.1002/bdr2.70104 (PMC13429490; doi:10.1002/bdr2.70104)
Supplement: Supplementary file 1 — Table S1: Relative organ weights (g/100 g body weight) of female offspring at PND 22. Table S2: Relative organ weights (g/100 g body weight) of male offspring at PND 22. Table S3: Hematological parameters of female offspring at PND22. Table S4: Hematological parameters of male offspring at PND22. [file BDR2-118-e70104-s001.docx]

**SUPPLEMENTARY MATERIAL**

**Results**

**Table S1.** Relative organ weights (g/100 g body weight) of female offspring at PND 22.

| **Parameters** | **Experimental groups** | | | |
| --- | --- | --- | --- | --- |
|  | **CTRL** | **APAP** | **ASA** | **NIM** |
| Body weight (g) | 50.09 ± 0.93 | 50.75 ± 1.9 | 49.53 ± 1.76 | 38.82 ± 1.69 |
| Right ovary | 0.022 ± 0.001 | 0.020 ± 0.001 | 0.027 ± 0.001 | 0.022 ± 0.00 |
| Total ovaries | 0.045 ± 0.003 | 0.041 ± 0.003 | 0.052 ± 0.003 | 0.050 ± 0.003 |
| Uterus | 0.056 ± 0.003 | 0.066 ± 0.005 | 0.061 ± 0.004 | 0.055 ± 0.003 |
| Spleen | 0.487 ± 0.023 | 0.459 ± 0.013 | 0.464 ± 0.018 | 0.471 ± 0.023 |
| Adrenal gland | 0.015 ± 0.000 | 0.014 ± 0.000 | 0.016 ± 0.000 | 0.017 ± 0.001 |
| Kidney | 0.607 ± 0.012 | 0.598 ± 0.011 | 0.612 ± 0.011 | 0.608 ± 0.013 |
| Liver | 4.232 ± 0.075 | 4.217 ± 0.074 | 4.131 ± 0.048 | 4.218 ± 0.097 |
| Thyroid | 0.009 ± 0.001 | 0.009 ± 0.001 | 0.010 ± 0.001 | 0.009 ± 0.001 |

Values are expressed as mean ± standard error of the mean (SEM). The litter was used as the statistical unit of comparison. n = 10 litters/group. ANOVA followed by Dunnett’s post hoc test. CTRL, control; APAP, paracetamol/acetaminophen; ASA, aspirin; NIM, nimesulide.

**Table S2.** Relative organ weights (g/100 g body weight) of male offspring at PND 22.

| **Parameters** | **Experimental groups** | | | |
| --- | --- | --- | --- | --- |
|  | **CTRL** | **APAP** | **ASA** | **NIM** |
| Body weight (g) | 48.71 ± 1.58 | 50.83 ± 2.32 | 49.26 ± 1.47 | 52.81 ± 1.73 |
| Liver | 4.375 ± 0.146 | 4.103 ± 0.147 | 4.202 ± 0.090 | 4.117 ± 0.124 |
| Kidney | 0.608 ± 0.019 | 0.585 ± 0.007 | 0.589 ± 0.009 | 0.600 ± 0.015 |
| Adrenal gland | 0.015 ± 0.001 | 0.014 ± 0.001 | 0.015 ± 0.001 | 0.014 ± 0.001 |
| Spleen | 0.464 ± 0.004 | 0.473 ± 0.035 | 0.413 ± 0.022 | 0.461 ± 0.025 |
| Thyroid | 0.008 ± 0.001 | 0.009 ± 0.001 | 0.007 ± 0.001 | 0.008 ± 0.001 |
| Testis | 0.240 ± 0.010 | 0.235 ± 0.007 | 0.240 ± 0.005 | 0.232 ± 0.005 |
| Epididymis | 0.036 ± 0.002 | 0.033 ± 0.002 | 0.031 ± 0.001 | 0.033 ± 0.001 |
| Vas deferens | 0.018 ± 0.000 | 0.015 ± 0.000 | 0.017 ± 0.000 | 0.018 ± 0.000 |
| Seminal vesicle | 0.023 ± 0.001 | 0.025 ± 0.003 | 0.031 ± 0.002* | 0.027 ± 0.001 |
| Prostate | 0.038 ± 0.004 | 0.044 ± 0.004 | 0.046 ± 0.003 | 0.040 ± 0.002 |

Values are expressed as mean ± standard error of the mean (SEM). The litter was used as the statistical unit of comparison. n = 10 litters/group. One-way ANOVA followed by Dunnett’s post hoc test. *p < 0.05 compared with the control group. CTRL, control; APAP, paracetamol/acetaminophen; ASA, aspirin; NIM, nimesulide.

**Table S3.** Hematological parameters of female offspring at PND22.

| **Parameters** | **Experimental groups** | | | |
| --- | --- | --- | --- | --- |
|  | **CTRL** | **APAP** | **ASA** | **NIM** |
| Platelets (10³/µL) | 843.1±71.03 | 744.7±48.56 | 688.5±100.1 | 644.6±83.38 |
| #Erythrocytes (10⁶/µL) | 5.15 (4.91-5.90) | 5.28 (4.68-6.21) | 5.47 (4.84-6.28) | 5.25 (4.72-6.83) |
| Hematocrit (%) | 33.18±1.23 | 34.93±1.76 | 34.36±1.71 | 35.53±3.15 |
| Leukocytes (10³/µL) | 5.23±0.44 | 5.36±0.21 | 5.09±0.54 | 4.99±0.58 |
| Hemoglobin (g/dL) | 10.72±0.31 | 10.91±0.63 | 10.78±0.57 | 11.02±0.68 |
| MCV (fL) | 62.61±1.08 | 64.91±1.27 | 61.86±0.94 | 62.4±1.35 |
| MCH (pg) | 19.19±0.53 | 20.23±0.57 | 19.48±0.41 | 19.36±0.47 |
| MCHC (g/dL) | 30.56±0.49 | 31.14±0.34 | 31.47±0.34 | 31.03±0.39 |
| Lymphocytes (%) | 74.78±1.20 | 75.33±1.07 | 76.0±1.93 | 75.78±1.37 |
| Neutrophils (%) | 20.56±1.0 | 20.33±0.91 | 20.89±1.78 | 20.22±1.31 |
| Monocytes (%) | 2.44±0.47 | 3.33±0.73 | 1.78±0.40 2.56±0.5 | |

Data are expressed as mean ± SEM. The litter was used as the statistical unit of comparison. n = 10 litters/group. One-way ANOVA followed by Dunnett’s post hoc test. Values marked with # are expressed as median and interquartile range (Q1–Q3) and were analyzed by Kruskal–Wallis followed by Dunn’s post hoc test. CTRL, control; APAP, paracetamol/acetaminophen; ASA, aspirin; NIM, nimesulide.

**Table S4.** Hematological parameters of male offspring at PND22.

| **Parameters** | **Experimental groups** | | | |
| --- | --- | --- | --- | --- |
|  | **CTRL** | **APAP** | **ASA** | **NIM** |
| Platelets (10³/µL) | 874.0±27.34 | 821.8±103.8 | 941.3±50.02 | 1020±56.42 |
| Erythrocytes (10⁶/µL) | 4.93±0.14 | 5.60±0.33 | 5.21±0.17 | 4.77±0.07 |
| Hematocrit (%) | 30.38±0.81 | 36.21±1.91* | 32.25±1.31 | 30.97±0.90 |
| Leukocytes (10³/µL) | 5.49±0.28 | 4.7±0.45 | 5.12±0.52 | 5.88±0.59 |
| Hemoglobin (g/dL) | 9.28±0.23 | 10.28±0.96 | 9.97±0.43 | 9.63±0.33 |
| MCV (fL) | 61.73±1.13 | 64.99±2.14 | 61.88±1.02 | 64.77±1.39 |
| MCH (pg) | 18.86±0.33 | 19.53±0.72 | 19.15±0.43 | 20.13±0.47 |
| #MCHC (g/dL) | 30.6 (30.3-30.88) | 30.3 (30.0-31.3) | 30.8 (30.0-31.1) | 30.40 (29.85-31.3) |
| Lymphocytes (%) | 73.71±1.15 | 77.67±2.14 | 76.57±1.29 | 77.0±4.51 |
| Neutrophils (%) | 22.14±0.96 | 19.83±2.63 | 20.29±1.51 | 18.33±3.84 |
| Monocytes (%) | 2.86±0.80 | 2.33±0.95 | 1.86± 0.34 3.0±0.0 | |

Data are expressed as mean ± SEM. The litter was used as the statistical unit of comparison. n = 10 litters/group. One-way ANOVA followed by Dunnett’s post hoc test. Values marked with # are expressed as median and interquartile range (Q1–Q3) and were analyzed by Kruskal–Wallis followed by Dunn’s post hoc test. *p < 0.05 compared with the control group. CTRL, control; APAP, paracetamol/acetaminophen; ASA, aspirin; NIM, nimesulide.
